# Supplementary material for: Ferrocene-Based Conjugated Microporous Polymers Derived from Yamamoto Coupling for Gas Storage and Dye Removal
Source: Polymers (Basel). 2020 Mar 24;12(3):719. doi: 10.3390/polym12030719 (PMC7183264; doi:10.3390/polym12030719)
Supplement: Supplementary file 1 [file polymers-12-00719-s001.pdf]

Supplementary Material

Ferrocene-based Conjugated Microporous Polymers Derived fromYamamoto Coupling for Gas Storage and Dye Removal

Zhiqiang Tan[1], Huimin Su[1], Yiwen Guo[1], Huan Liu[1], Bo Liao[1], Abid Muhammad Amin[2] and Qingquan Liu[1]*

[1] School of Materials Science and Engineering; Hunan Provincial Key Laboratory of Advanced Materials for New Energy Storage and Conversion; Hunan Provincial Key Laboratory of Controllable Preparation and Functional Application of Fine Polymers, Hunan University of Science and Technology, Xiangtan, 411201, China
[2] Department of Chemistry, University of Sahiwal, Sahiwal, Pakistan
* Corresponding Author. E-mail: qqliu@hnust.edu.cn

Synthesis and characterization of TBPP and TBPM

1. Synthesis of 5,10,15,20-Tetrakis(4-bromophenyl)porphyrin(TBPP)

A solution of bromobenzaldehyde (1.6 g, 8.65 mmol) in propionic acid (50 mL) was heated to 135°C. Pyrrole (0.6 mL, 8.65 mmol) and glacial acetic acid(1.2 ml) were slowly added into the mixture. The mixture was refluxed for 1.5 h. A black suspension was obtained and moved to refrigerator overnight. The crude products were gotten by filtration and washed with methanol until the filtrate was colorless. Finally, the purple powder were dried in vacuo. ^1H NMR (500 MHz, $CDCl_3$, Fig.1) δ 8.84 (s, 6H), 8.06 (d, J = 8.2 Hz, 6H), 7.90 (dd, J = 15.2, 8.5 Hz, 6H), -2.88 (s, 2H).

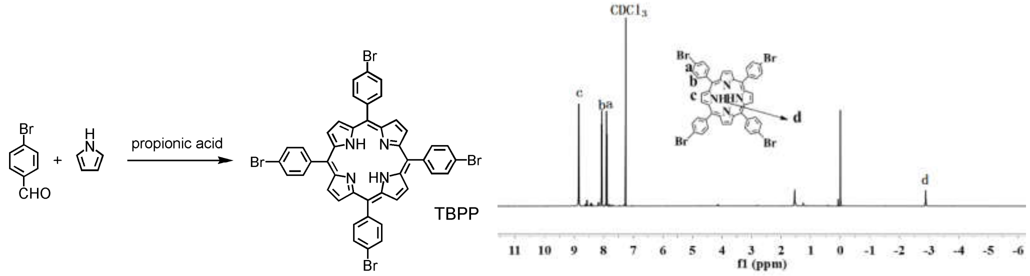

Figure S1. Synthetic route and ^1H NMR spectrum of 5,10,15,20-Tetrakis(4-bromophenyl) porphyrin.

2. Synthesis of Tetra(p-bromophenyl)methane(TBPM)

A mixture of triphenylmethanol (1.3 g, 5 mmol), aniline (0.7 mL, 7.5 mol), concentrated hydrochloric acid (2.5 mL) and glacial acetic acid (15.0 mL) were refluxed at 60 °C for 48 h. The precipitates were filtered and re-dissolved in ethanol (75.0 ml), which was then cooled to -15 °C. Concentrated sulfuric acid (5.0 ml) and isoamyl nitrite(3.0 ml) were added and allowed to react for 1h. Hypophosphorous acid(6.0 ml, 60%) was added to the mixture until no gas was produced. The products were filtered, and washed with distilled water and ethanol, respectively. Finally, the brown products (tetraphenylmethane) were dried in vacuo (1.9 g, 58.99%).

Tetraphenylmethane(1.8 g, 5.6 mmol) was added to pure bromine (6.0 ml, 125.0 mmol) at room temperature, and the mixture was cooled to -75 ^{o}C and stirred for 2 h. After 15.0 ml ethanol was added, and the mixture was naturally warmed to room temperature and stirred overnight. The crude products were filtered and washed with sodium bisulfite solution. The crude products were recrystallized for twice in co-solvent of dichloromethane and ethanol, and yellow TBPM was obtained. ^{1}H NMR (500 MHz, $CDCl_3$, Fig.1) δ 7.41 (dd, J = 18.1, 9.2 Hz, 2H), 7.00 (dd, J = 8.6, 4.1 Hz, 2H).

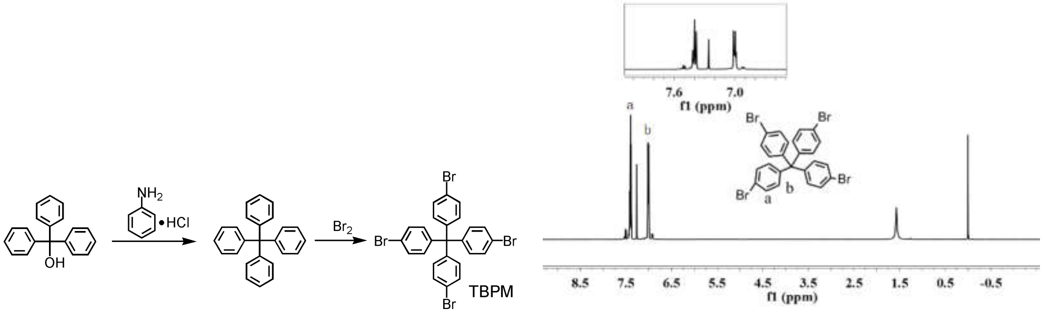

Figure S2. Synthetic route and ^{1}H NMR spectrum of Tetra(p-bromophenyl)Methane (TBPM).

3. Gas capture performance of FcCMPs and other porous polymers

Table S1 Summary of Gas capture performance in porous organic polymers

Materials	CO_2 adsorption capacity(mmol/g)	H_2 adsorption capacity(mmol/g)	CH_4 adsorption capacity(mmol/g)	BET Surface area (m^2/g)	Reference
FcCMP-1	2.18	5.5	0.69	638	This work
FcCMP-2	1.61	3.5	0.64	422	This work
COFs					
ILCOF-1	1.36	6.5	0.64	2723	[26]
TDCOF-5	2.1	8.0	0.51	3832	[27]
COF-10	1.25	4.2	0.37	1760	[28]
COF-102	1.65	6.0	0.65	3620	[28]
CMPs					
BCMP-2	1.69	—	—	911	[29]
TCMP-0	2.38	—	—	963	[30]
Fc-CMP-1	1.45	—	0.61	519	[30]
HP$_E$-CMP	3.58	—	0.45	662	[31]
CMP	—	8.0	—	955	[32]
Li-CMP	—	30.5	—	795	[32]
POPs					
FNOPs-1	3.78	6.35	2.17	752	[11]
MPOP-3	3.36	4.8	0.99	1026	[20]
FPOP-2	1.44	7.0	—	954	[33]
FMAP-1	3.84	8.75	—	875	[17]
CPOP-9	4.14	12.2	—	2440	[34]
BLP-12	2.91	9.65	0. 81	2244	[35]

Adsorption conditions of CO_2 and CH_4: 1bar/273K; Adsorption conditions of H_2: 1bar/77K
